# Relationships between plasma lipidomic profiles and brown adipose tissue density in humans

**DOI:** 10.1038/s41366-020-0558-y

**Published:** 2020-03-03

**Authors:** Sayuri Fuse, Masahiro Sugimoto, Yuko Kurosawa, Miyuki Kuroiwa, Yasuko Aita, Atsumi Tomita, Eri Yamaguchi, Riki Tanaka, Tasuki Endo, Ryotaro Kime, Takafumi Hamaoka

**Affiliations:** 1 Department of Sports Medicine for Health Promotion, Tokyo Medical University, Tokyo, Japan; 2 Health Promotion and Preemptive Medicine, Research and Development Center for Minimally Invasive Therapies, Tokyo Medical University, Tokyo, Japan

**Keywords:** Translational research, Public health

## Abstract

**Background/objectives:**

The thermogenic function of brown adipose tissue (BAT) is generally activated in winter and tightly regulated through various metabolic processes. However, the mechanisms mediating these changes have not been elucidated in humans. Here, we investigated the relationships between BAT density (BAT-d) and lipid metabolites in plasma from men and women in the winter and summer.

**Subjects/methods:**

In total, 92 plasma samples were obtained from 23 men and 23 women, aged 21–55 years, on two different occasions (summer and winter). Lipid metabolites were comprehensively quantified using liquid chromatography-time-of-flight-mass spectrometry. BAT-d was evaluated by measuring total hemoglobin concentrations in the supraclavicular region using near-infrared time-resolved spectroscopy. Anthropometric parameters, such as the percentage of whole body fat and visceral fat area (VFA), were evaluated. Factors influencing BAT-d were investigated by univariate and multivariate regression analyses.

**Results:**

A variety of metabolite peaks, such as glycerophospholipids (168 peaks), steroids and derivatives (78 peaks), fatty acyls (62 peaks), and glycerolipids (31 peaks), were detected. Univariate regression analysis, corrected by false discovery rate to yield *Q* values, revealed significant correlations in BAT-d and phosphatidylethanolamine (PE(46:2), *r* = 0.62, *Q* = 4.9 × 10^−2^) in the summer, androgens (*r* = 0.75, *Q* = 7.0 × 10^−3^) in the winter, and diacylglycerol (DG(36:1), *r* = −0.68, *Q* = 4.9 × 10^−2^) in the summer in men, but not in women. Multivariate regression analysis in the winter revealed a significant correlation between BAT-d and plasma androgens (*P* = 5.3 × 10^−5^) in men and between BAT-d and VFA (*P* = 2.2 × 10^−3^) in women.

**Conclusions:**

Certain lipids in plasma showed unique correlations with BAT-d depending on sex and season. BAT-d showed a specific correlation with plasma androgens in men in the winter.

## Introduction

Brown adipose tissue (BAT) functions in adaptive thermogenesis in response to cold exposure or nutritional intake [1–4]. Human BAT activity is deteriorated with advancing age and related to lower body adiposity [3, 4]. BAT enhances glucose metabolism not only in healthy individuals [1–5] but also in obese individuals [6] and patients with type 2 diabetes [7]. BAT activity is higher in winter [3, 4] and increased during 4 and 6 weeks after the ambient temperature decreases to lower than 4 or 5 °C, as indicated by quantitative analyses [8]. The activity and/or amount of human BAT are thought to be modulated by a genetic preposition and environmental factors. Some single nucleotide polymorphisms in specific genes are known to be related to BAT activity. For example, uncoupling protein 1 and β3-adrenergic receptor substitutions, which accelerate age-related decreases in BAT activity, are associated with visceral fat accumulation during aging [9, 10]. Although cold exposure is one of the strongest environmental stimuli enhancing human BAT capacity, it is difficult to monitor the amount of cold exposure for each individual during daily life. Thus, further studies are needed to identify biomolecules associated with BAT activation in humans.

Quantitative lipid analyses of blood samples by direct infusion mass spectrometry (MS) have been used to study lipid metabolism with high sensitivity and broad range profiling relating to BAT [11, 12]. Previous lipidomic analyses have reported the specific characteristics of BAT and white adipose tissue (WAT) in response to exercise or cold exposure [12–15]. BAT activity and/or volume are related to several lipid metabolites, such as the concentration of lysophosphatidylcholine-acyl (LysoPC-acyl) C16:0 in humans [16], and the concentration of phosphatidylethanolamine (PE) in the BAT and WAT was reduced by a high-fat diet in mice [12].

Near-infrared time-resolved spectroscopy (NIR_TRS_) has been used to noninvasively quantify tissue total hemoglobin concentrations (total-Hb). Among these NIR_TRS_ parameters, total-Hb concentrations in the supraclavicular region ([total-Hb]_sup_) have been investigated for evaluating BAT density (BAT-d) as a potential index of blood volume (or tissue vasculature density) [17]. Notably, vascular density is higher in BAT than in WAT. The [total-Hb]_sup_ under both thermoneutral and cold conditions was positively correlated with parameters determined by cold-induced ^18^F-fluorodeoxyglucose (FDG)-positron emission tomography (PET) used in conjunction with computed tomography (CT) (^18^FDG-PET/CT) in the supraclavicular region, which potentially contains BAT deposits, but not in the deltoid muscle region control site [17]. Moreover, significant correlations were reported between cold-induced thermogenesis (CIT), an indicator for BAT activity [3, 4], and [total-Hb]_sup_ in winter [18]. Thus, NIR_TRS_ could be a reasonable alternative to ^18^FDG-PET/CT, which has several limitations, including enormous instrumentation costs, ionizing radiation exposure, and acute cold exposure [19].

Here, we hypothesized that certain lipids in the plasma determined by liquid chromatography-time-of-flight MS may show unique correlations with BAT-d depending on sex and season. Thus, the purpose of this study was to investigate the relationships between BAT-d determined by NIR_TRS_ and lipid metabolites in the plasma in men and women in winter and summer.

## Materials and methods

### Subjects and samples

Healthy volunteers with BAT-d values ≥ 74.0 µM were recruited from a group of individuals 20–59 years of age (*n* = 52), whom we previously evaluated in the winter of 2016 in the Tokyo area. Among these individuals, 23 volunteers with higher BAT-d values agreed to participate in the study in the summer of 2017, and 21 participated in the winter of 2018. Age-, sex-, and body mass index (BMI)-matched volunteers with lower BAT-d values of ≤70.0 µM (*n* = 69) were recruited; 29 volunteers agreed to participate in the study in the summer of 2017, and 25 participated in the winter of 2018. Thus, we tested 23 men and 23 women in the winter and summer (participant ages: 21–55 years), yielding 92 plasma samples. After the participants arrived at the laboratory, the following parameters were measured: body height, body weight, percentage of body fat (%BF), visceral fat area (VFA), systolic blood pressure (SBP), diastolic blood pressure (DBP), heart rate (HR), BAT-d, and lipid metabolites in blood plasma. Room temperature was regulated from 23 to 27 °C using an air conditioner. The study design and protocols were approved by the institutional review board of Tokyo Medical University (approval no. 2017-199), in accordance with the ethical principles contained in the Declaration of Helsinki. Written informed consent was obtained from all participants.

### Measurement of anthropometric and circulatory parameters

BMI was calculated as follows: body weight in kilograms divided by the square of height in meters (kg/m^2^). The %BF was estimated by the multifrequency bioelectric impedance method (Inbody 720; InBody Japan, Tokyo, Japan). VFA, at the abdominal level of L4, was estimated using a bioelectrical impedance analysis (EW-FA90; Panasonic, Osaka, Japan). SBP, DBP, and HR were measured using an automated sphygmomanometer (HBP-9020; Omron Healthcare, Kyoto, Japan).

### BAT-d measurements

BAT-d, evaluated by the [total-Hb]_sup_ using NIR_TRS_ (TRS-20; Hamamatsu Photonics K.K., Hamamatsu, Japan), was measured for 1 min at 23–27 °C. The probes were placed on the skin of the supraclavicular region, which may contain BAT, and participants were required to remain in a sitting position during the measurements, as previously described [8, 17, 20–22]. Compared with visible light wavelengths, NIR wavelengths (700–3000 nm) show less scattering and, consequently, better penetration into biological tissue. However, light absorption by water limits tissue penetration above 900 nm wavelength; thus, 650–900-nm range is suitable for measurements [23]. Accordingly, we used NIR wavelengths of 760, 800, and 830 nm to evaluate oxyhemoglobin concentrations, deoxyhemoglobin concentrations, and total-Hb concentrations. With the 3-cm probe used in this study, light can reach a mean depth of 2 cm [24], where BAT is potentially located [25].

The tissue was illuminated using a 200-µm core diameter optical fiber by the pulsed light generated from ps light pulses, with 100 ps full width at half-maximum, a 5-MHz repetition rate, and an average power of 80 µW for each wavelength. The emitted photons penetrated the tissue and were reflected to a 3-mm diameter optical bundle fiber, through which they were sent to a photomultiplier tube for single-photon detection and a signal processing circuit for time-resolved measurement. Using the nonlinear least-squares method, the digitized temporal profile data from an in vitro sample or tissue was fitted with a theoretical temporal profile, derived from the analytical solution of the photon diffusion theory with a semi-infinite homogeneous reflectance model. After convolution with the instrumental response function, so the time response of the instrument itself could be compensated for, the absorption coefficient values and reduced scattering coefficient values at 760, 800, and 830 nm were obtained using the least-squares fitting method. Then, the absolute total-Hb concentration was calculated as the sum of oxyhemoglobin and deoxyhemoglobin concentrations [8, 17, 23]. The NIR_TRS_ system collected data every 10 s. The coefficient of variation for repeated measurements of the total-Hb concentration was 4.9% [17].

Our previous study indicated that a cutoff value of 74.0 μM [total-Hb]_sup_ for distinguishing BAT negativity from BAT positivity (evaluated by FDG-PET/CT) resulted in the best prediction accuracy of 82.8%, with a sensitivity of 75.0%, specificity of 100%, positive predictive value of 100%, and negative predictive value of 64.3% [17].

### Metabolic analysis

#### Chemicals

LC-MS-grade methanol, 2-propanol, acetonitrile, high-performance liquid chromatography-grade chloroform, and 1 M ammonium formate solution were obtained from Wako (Osaka, Japan). Three reagents were used as internal standards (ISs): *N*-lauroyl-d-erythro-sphingosylphosphorylcholine was from Avanti Polar Lipids (Alabaster, AL, USA), etodolac was from Wako, and 1,2-dimyristoyl-sn-glycero-3-phosphocholine was from Tokyo Chemical Industry (Tokyo, Japan). Water was purified on a Milli-Q system (Merck Millipore, Bedford, MA, USA).

#### Processing of human plasma

Blood samples from the median cubital vein were drawn by smooth venipuncture employing minimal stasis and stored in siliconized glass tubes with ethylenediaminetetraacetic acid (VenoJect; Terumo, Tokyo, Japan). Plasma for nontargeted metabolomic profiling was obtained after centrifugation at 3000 × *g* at 4 °C for 10 min and stored at −80 °C until analysis of lipid metabolites. Human plasma (30 μL) was mixed with 2-propanol (270 μL) containing 0.222 μM ISs (*N*-lauroyl-D-erythro-sphingosylphosphorylcholine, etodolac, 1,2-dimyristoyl-sn-glycero-3-phosphocholine). After centrifugation at 15,780 × *g* for 10 min at 4 °C, supernatants were transferred to another tube and vacuum dried for 1 h at room temperature (VC-96W; TAITEC, Saitama, Japan). The samples were mixed with 150 μL methanol, chloroform, and Milli-Q water at a volume ratio of 2:4:1 (v:v:v) and centrifuged at 15,780 × *g* for 5 min at 4 °C. Finally, 100 μL of the supernatant was used for LC-TOF-MS analyses.

#### LC conditions

The LC system was an Agilent Technologies 1290 Infinity instrument (Agilent Technologies, Santa Clara, CA, USA), consisting of a HiP sampler, quaternary pump, and column compartment. Chromatographic separation was performed using an ACQUITY UPLC HSS T3 column (2.1 i.d. × 50 mm, 1.7 mm; Waters, Milford, MA, USA) at 45 °C. The mobile phase, consisting of solvent A (0.5 mM ammonium formate in water, methanol, and acetonitrile at a volume ratio of 3:1:1) and solvent B (0.5 mM ammonium formate in 2-propanol), was delivered at a flow rate of 0.3 mL/min. The gradient elution is listed in Table 1S. The total run time for LC-MS analysis was 25 min/sample.

#### MS conditions

MS detection was conducted on an Agilent Technologies G6230B TOF-MS. The samples were analyzed in positive and negative ion mode. Instrument parameters were set as follows: drying gas temperature at 350 °C, drying gas flow at 12 L/min, nebulizing at 55 psig, Vcap at 4000 V (positive) and 3500 V (negative), fragmentation at 180 V, skimming at 90 V, Octopole RF Peak at 230 V (positive) and 210 V (negative), mass range of 50–1700 *m/z*, and scan rate of 1.00 spectrum/s. Agilent MassHunter Qualtative Analysis software (version B.08.00; Agilent Technologies) was used for data processing.

#### Signal selection to be analyzed

Processed plasma samples were diluted by adding methanol, chloroform, and Milli-Q water at a volume ratio of 2:4:1 containing 2 μM ISs (*N*-lauroyl-d-erythro-sphingosylphosphorylcholine, etodolac, 1,2-dimyristoyl-sn-glycero-3-phosphocholine). In total, four samples (diluted ×1, ×1/2, ×1/4, and ×1/8) were analyzed by LC-TOF-MS. Thus, 1771 theoretical *m/z* values were yielded from the metabolites listed in the lipid metabolism category in the Kyoto Encyclopedia of Genes and Genomes (KEGG) ligand database and fatty acids, phospholipids, neutral lipids, and sphingolipids with all possible combinations of fat chains. The peaks were extracted by MassHunter (matching tolerance: 10 ppm, adduct ion: +H, +NH_4_ (positive) –H, +HCOO (negative), height count: 1000 or more, charge number range: 1–2). The peaks were matched across the four samples according to *m/z* values and retention times. In total, 159 peaks were detected in only one of the four samples, 153 peaks were detected in two of the four samples, 189 peaks were detected in three of the four samples, and 149 peaks were detected in all samples. Peaks with different adduct ions at the same retention time adopted those with large areas. For peaks detected in three of the four samples, peaks showing high linearity (*R* = 0.8, *n* = 3, 4) among peak areas and dilution rates were selected for further analysis. Peaks detected in two of the four samples were also selected. In total, 398 peaks were analyzed.

### Data analysis and statistical analysis

Data processing for lipid metabolites was conducted using MassHunter Qualitative Analysis software (Agilent) according to typical procedures [26]. Possible metabolite names were assigned by comparing observed and theoretical *m/z* values within 10 ppm. As candidate metabolites, 1771 theoretical *m/z* values were calculated for fatty acids, phospholipids, neutral lipids, sphingolipids, and metabolites in the lipid metabolism category of the KEGG Pathway Database. Peaks were integrated automatically, and the detected peaks were then manually curated. Each peak area was divided by those of *N*-lauroyl-d-erythro-sphingosylphosphorylcholine to generate relative areas, and all samples were measured in a single batch for eliminating unexpected bias caused by MS sensitivity.

Spearman correlation analysis was used for evaluating the correlations among metabolites and other observations, such as BAT-d. *P* values were corrected by false discovery rates to yield *Q* values, considering multiple independent tests (GraphPad Prism ver. 7.03; SAS Institute Inc., Cary, NC, USA). Thereafter, Mev TM4 (ver. 4.9.1) was used to show the rank order of correlations between BAT-d and metabolites [27].

The primary outcome variable for estimating sample size was a seasonal change in BAT-d. The primary hypothesis was tested against two-directional alternatives with a 5% significance level. The objective was to achieve at least 80% power to detect a minimum final difference in the seasonal change in BAT-d. This resulted in a net sample size of at least 35 participants.

Anthropometric and circulatory parameters were expressed as means ± standard deviations (SDs). If normality was detected by the Shapiro-Wilk test, paired *t*-tests were used to test for seasonal differences in anthropometric and circulatory parameters. If normality was not detected, Wilcoxon signed rank tests were conducted. In addition, to examine sex differences, Welch *t*-tests or Mann-Whitney tests were used after a test for normality was performed for each parameter. *P* values and *Q* values were determined for evaluating the correlations between BAT-d and each metabolite and between indicators of body adiposity (%BF and VFA) and each metabolite. Univariate and multivariate regression analyses were conducted as follows: BAT-d as a dependent variable with age, BMI, %BF, VFA, and each plasma metabolite as independent variables; and each metabolite as a dependent variable with sex, season, age, and BMI as independent variables. Three-way analysis of variance was used to test the interactions (BAT-d group × season × sex) and main effects (BAT-d group, season, and sex). To divide BAT-d into two groups (high and low), the cutoff value obtained in a previous study in winter [17] was used (high, [total-Hb]_sup_ ≥ 74.0 μM; low < 74.0 μM). To conduct the analyses, categorical variables were set at “0” for the low group and “1” for the high group, “0” for summer and “1” for winter, and “0” for women and “1” for men. Results with *P* values of <0.05 were considered statistically significant. These analyses were conducted using SPSS (IBM SPSS Statistics 25; IBM Japan, Tokyo, Japan).

## Results

### Participant profiles

Among all participants, we found that age, %BF, SBP, and BAT-d were significantly increased from summer to winter. In men, age, body weight, BMI, %BF, and VFA were found to be higher in winter than in summer, whereas in women, age and BAT-d were significantly increased from summer to winter (Table 1).

**Table 1:** Participant profiles.

	Summer			Winter		
	All (*n* = 46)	Men (*n* = 23)	Women (*n* = 23)	All (*n* = 46)	Men (*n* = 23)	Women (*n* = 23)
Age (year)	40.8 ± 8.2	40.1 ± 8.3	41.4 ± 8.3	41.4 ± 8.3*	40.8 ± 8.3*	42.0 ± 8.4*
Height (cm)	167.2 ± 7.6	173.1 ± 4.7	161.3 ± 4.9^†^	167.3 ± 7.7	173.3 ± 4.8	161.3 ± 4.9^†^
Body weight (kg)	61.5 ± 10.8	67.9 ± 10.0	55.0 ± 7.1^†^	61.8 ± 11.2	68.9 ± 10.0*	54.7 ± 7.0^†^
BMI (kg/m^2^)	21.9 ± 3.0	22.7 ± 3.4	21.1 ± 2.4	22.0 ± 3.1	23.0 ± 3.3*	21.0 ± 2.4^†^
%BF (%)	23.3 ± 8.0	18.4 ± 7.4	28.1 ± 5.2^†^	24.3 ± 7.8*	19.8 ± 7.2*	28.8 ± 5.4^†^
VFA (cm^2^)	45.7 ± 32.8	57.0 ± 42.1	34.9 ± 14.8	48.9 ± 35.5	62.9 ± 43.0*	34.9 ± 17.9^†^
SBP (mmHg)	106.4 ± 13.7	111.7 ± 11.5	101.1 ± 13.8^†^	109.5 ± 15.1*	114.4 ± 13.4	104.7 ± 15.4^†^
DBP (mmHg)	69.0 ± 12.1	73.0 ± 12.1	65.0 ± 10.9^†^	70.5 ± 12.7	73.2 ± 11.9	67.9 ± 13.2
HR (bpm)	69.0 ± 9.1	69.3 ± 7.9	68.7 ± 10.4	70.2 ± 10.2	71.3 ± 10.2	69.2 ± 10.3
BAT-d (µM)	63.3 ± 20.6	69.0 ± 24.7	57.6 ± 13.7	69.4 ± 20.9*	70.8 ± 23.1	68.0 ± 18.8*

Values are means ± SDs.

*BMI* body mass index, %*BF* percentage of body fat, *VFA* visceral fat area, *SBP* systolic blood pressure, *DBP* diastolic blood pressure, *HR* heart rate, *BAT-d* brown adipose tissue density.

*P < 0.05 summer versus winter; ^†^*P* < 0.05 men versus women.

### Metabolomic data

In this study, various peaks were detected, such as glycerophospholipids (168 peaks), steroids and derivatives (78 peaks), fatty acyls (62 peaks), and glycerolipids (31 peaks). Among nontargeted analyses of samples with four different dilutions, 398 peaks were detected in at least two samples and, of these, 189 peaks showing highly positive correlations among peak area and inverse dilutions (*R* = 0.8) were analyzed for subsequent statistical analyses.

### Correlations between metabolites and BAT-d

**Fig. 1:**
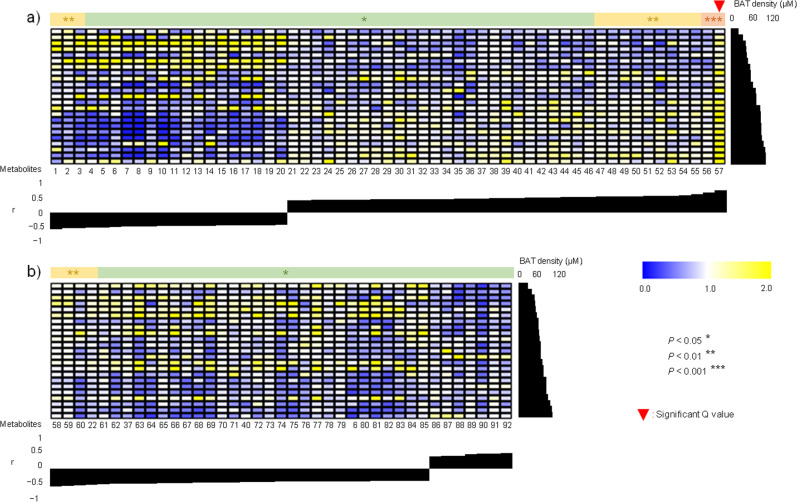
Relationships between plasma metabolites and brown adipose tissue density (BAT-d) in winter. **a** In winter, among metabolites showing correlations (*P* < 0.05) with BAT-d, only androgens (testosterone, androstanedione, dehydroandrosterone, dehydroepiandrosterone, or epitestosterone labeled as 57 in Table 2S) showed significant positive correlations at *Q* < 0.05 in men. **b** In women, no metabolites showed any significant correlations at *Q* < 0.05. *P* values were corrected by false discovery rate to yield *Q* values, for considering independent multiple tests. The levels of BAT-d are shown in the right edge of the heat map. The unit of the color scale was calculated by dividing the value of each substance by the average of values of corresponding substances for men and women both in summer and winter. For example, yellow (pale color) indicates the greater relative value of each metabolite, whereas blue (dark color) indicates the lower relative value of each metabolite. The number of the metabolites indicated below the heat map represents each substance, as listed in Table 2S. *r*, correlation coefficient between BAT-d and each metabolite (Color figure online).

Figure 1 depicts metabolites showing correlations with BAT-d (*P* < 0.05). In men, 37 and 20 metabolites showed positive and negative correlations (*P* < 0.05), respectively, and only one metabolite (a testosterone, androstanedione, dehydroandrosterone, dehydroepiandrosterone, or epitestosterone defined as androgens [#57] in Table 2S) showed a significant positive correlation (*Q* < 0.05) in winter. In women, 7 and 32 metabolites showed positive and negative correlations (*P* < 0.05), respectively, whereas no metabolites showed significant correlations at *Q* values of <0.05 in winter. In summer, two metabolites (PE(46:2), #55, and diacylglycerol (DG)(36:1), #93; Table 2S) showed significant correlations with BAT-d (*Q* < 0.05) only in men (Fig. 1S).

**Fig. 2:**
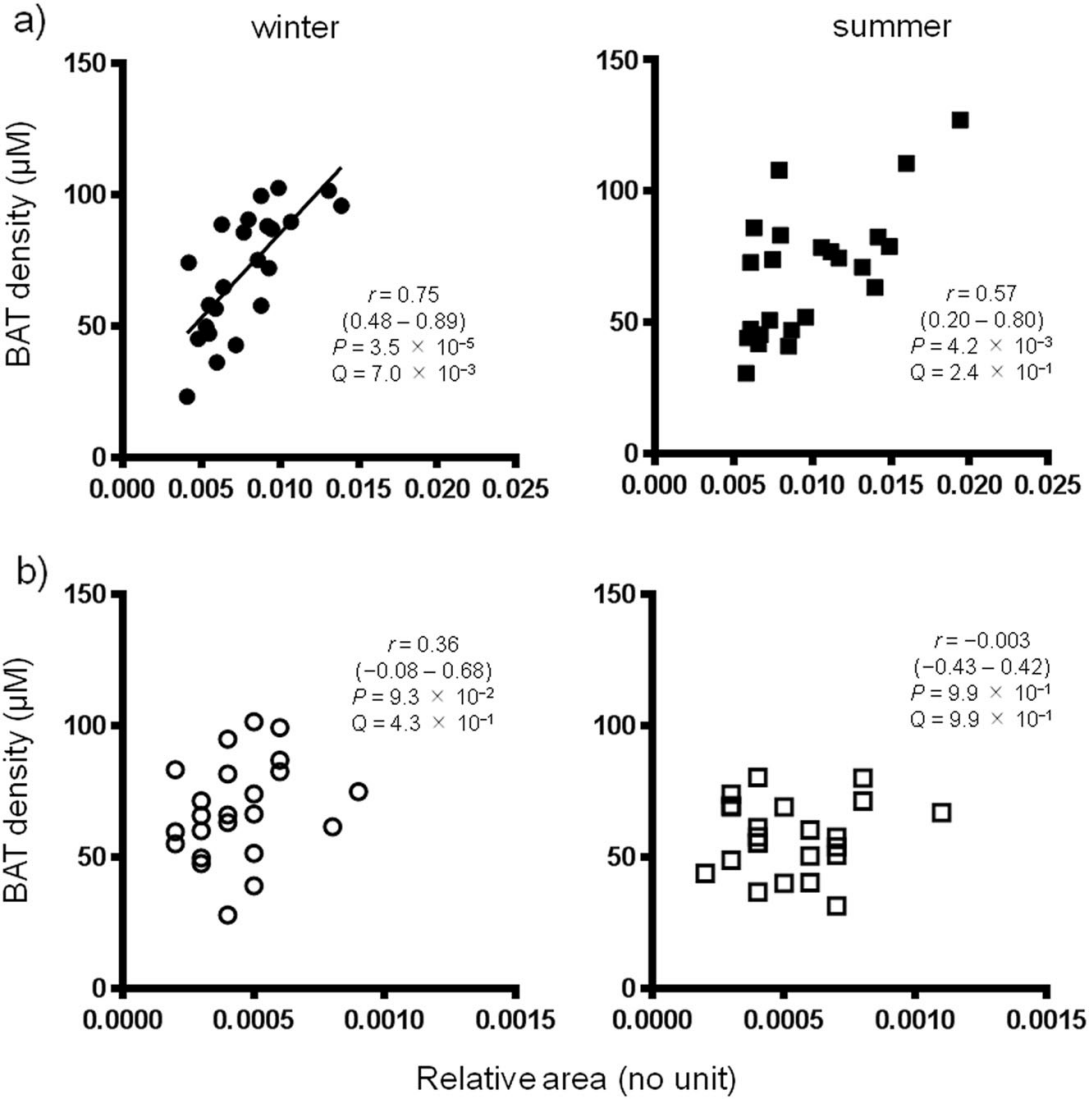
Relationships between androgens and brown adipose tissue density (BAT-d). **a** Relationships in men. **b** Relationships in women. *P* values were corrected by false discovery rate to yield *Q* values considering independent multiple tests.

Figure 2 shows the relationships between BAT-d and androgens. Interestingly, in men, BAT-d showed significant positive correlations with androgens both in summer and winter by Spearman analysis (*P* < 0.05), but did show a significant correlation in winter with a *Q* value cutoff of <0.05. BAT-d showed no seasonal changes in men. Combined data for summer and winter still revealed a significant (*Q* < 0.05) correlation between BAT-d and androgens (data not shown). In contrast, in women, there were no significant correlations. Similarly, in men, BAT-d showed significant positive correlations of BAT-d with PE(46:2) both in summer and winter by Spearman analysis (*P* < 0.05), but showed a significant correlation only in summer with a *Q* value cutoff of <0.05 (Fig. 2S). Moreover, the relationship between BAT-d and DG(36:1) was negatively correlated only in men in the summer (Fig. 3S).

Body adiposity parameters showed significant correlations with three metabolites (androgens, PE(46:2), and DG(36:1)) (Table 3S). There were significant (*Q* < 0.05) relationships in men between %BF and ceramide (Cer(18:1/24:0), #12 in Table 2S) both in summer and winter and between VFA and 3a, 7a-Dihydroxy-5b-cholextanoyl-CoA in winter. There was a significant (*Q* < 0.05) relationship in women between VFA and lysophosphatidylglycerol (LysoPG(p-24:5), #117 in Table 2S) in summer (Table 3S). We examined the repeatability of the data for six plasma metabolites as listed in Table 3S between summer and winter. The relationships for substances were all significant. The correlation coefficients ranged from 0.316 to 0.903. We also examined the correlations between season-dependent changes in metabolite levels (winter to summer) and BAT-d (in winter), as shown in Table 4S. We did not observe any significant correlations between BAT-d and any metabolites.

Next, we analyzed the associations of metabolites with BAT-d by pooling all data in Table 2. Multivariate regression analysis revealed significant correlations of androgens, PE, or DG with sex, age, and/or BMI and a significant correlation between androgens and season. These results indicated that only androgen exhibited seasonal fluctuations. There were main effects of BAT-d (high versus low; *P* = 2.3 × 10^−19^) and season (*P* = 1.9 × 10^−2^). In addition, there was an interaction between BAT-d (high versus low) and sex, but not an interaction between BAT-d (high versus low) and season (Fig. 4S). Multivariate regression analysis in winter revealed a significant correlation only between BAT-d and androgens (*P* = 5.3 × 10^−5^) in men and between BAT-d and VFA (*P* = 2.2 × 10^−3^) in women (Table 3).

**Table 2:** Multivariate regression analysis between lipid metabolites and sex, season, age, and body mass index (BMI) for all data from men and women and in summer and winter.

		All, *n* = 92				
		Univariate regression		Multivariate regression		
Dependent variables	Independent variables	*r*	*P*	B	Standardized *β*	*P*
Androgen	Sex	0.868	<0.001	0.009	0.926	<0.001
	Season	−0.119	2.5 × 10^−1^	−0.001	−0.109	2.4 × 10^−2^
	Age	−0.218	3.7 × 10^−2^	–	–	–
	BMI	0.153	1.5 × 10^−1^	−0.0003	−0.203	9.7 × 10^−5^
DG(36:1)	Sex	0.454	5.3 × 10^−6^	0.004	0.221	1.2 × 10^−2^
	Season	0.19	7.0 × 10^−2^	–	–	–
	Age	0.078	4.6 ×10^−1^	–	–	–
	BMI	0.391	1.2 ×10^−4^	0.002	0.527	2.5 × 10^−8^
PE(46:2)	Sex	−0.222	3.3 × 10^−2^	–	–	–
	Season	−0.058	5.8 × 10^−1^	–	–	–
	Age	0.214	4.0 × 10^−2^	0.00004	0.234	1.1 × 10^−2^
	BMI	−0.471	2.2 × 10^−6^	−0.0002	−0.514	1.7 × 10^−7^

*Androgens* testosterone, androstanedione, dehydroandrosterone, dehydroepiandrosterone, or epitestosterone, *DG* diacylglycerol, *PE* phosphatidylethanolamine.

**Table 3:** Multivariate regression analysis between brown adipose tissue density (BAT-d) and anthropometric and lipid metabolites in winter.

	Male, *n* = 23						Women, *n* = 23					
	Univariate regression			Multivariate regression			Univariate regression			Multivariate regression		
Dependent variable: BAT-d (µM)	*r*	95% CI	*P*	B	Standardized *β*	*P*	*r*	95% CI	*P*	B	Standardized *β*	*P*
Age (years)	−0.05^a^	−0.46 to 0.37	8.1 × 10^−1^	–	–	–	−0.32^b^	−0.65 to 0.12	1.4 × 10^−1^	–	–	–
BMI (kg/m^2^)	−0.70^b^	−0.87 to −0.40	1.8 × 10^−4^	–	–	–	−0.60^a^	−0.81 to −0.25	2.3 × 10^−3^	–	–	–
%BF (%)	−0.62^a^	−0.82 to −0.28	1.5 × 10^−3^	–	–	–	−0.54^a^	−0.78 to −0.17	7.7 × 10^−3^	–	–	–
VFA (cm^2^)	−0.71^b^	−0.88 to −0.38	4.7 × 10^−4^	–	–	–	−0.61^b^	−0.83 to −0.21	4.5 × 10^−3^	−0.70	−0.64	2.2 × 10^−3^
Androgens	0.75^b^	0.48 to 0.89	3.5 × 10^−5^	6550.48	0.78	5.3 × 10^−5^	0.36^b^	−0.08 to 0.68	9.3 × 10^−2^	–	–	–
DG(36:1)	−0.36^b^	−0.68 to 0.08	9.4 × 10^−2^	–	–	–	−0.41^b^	−0.71 to 0.02	5.3 × 10^−2^	–	–	–
PE(46:2)	0.62^b^	0.26 to 0.83	1.7 × 10^−3^	–	–	–	−0.02^b^	−0.44 to 0.41	9.4 × 10^−1^	–	–	–

*BMI* body mass index, %*BF* percentage of body fat, *Androgens* testosterone, androstanedione, dehydroandrosterone, dehydroepiandrosterone, or epitestosterone, *DG* diacylglycerol, *PE* phosphatidylethanolamine.

^a^Pearson’s product moment correlation coefficient.

^b^Spearman’s rank correlation coefficient.

## Discussion

In this study, we observed significant (*Q* < 0.05) positive correlations of BAT-d and PE(46:2) in summer and androgens in winter and negative correlation of BAT-d and DG(36:1) in summer only in men. Multivariate regression analysis in winter revealed a significant correlation between BAT-d and androgens in men and between BAT-d and VFA in women. Surprisingly, we found that in men, there was a relationship between BAT-d and androgens in winter, but no influence of an anthropometric parameter, BMI, which is generally associated with BAT-d.

To analyze BAT-specific metabolites involved in seasonal activation of BAT, we examined the correlations between season-dependent changes in metabolite levels (winter to summer) and BAT-d (in winter). However, we failed to observe any significant correlations between BAT-d and seasonal changes in any metabolites, including androgens, indicating that there were not specific metabolites involved in seasonal changes in BAT-d. We found significant correlations between BAT-d and BMI, %BF, and VFA in univariate regression analysis, although these correlations disappeared after adopting a stepwise multiple regression analysis in men, yielding only a significant correlation of BAT-d with androgens, and in women, except the significant correlation of BAT-d with VFA. When three metabolites (androgens, DG(36:1), and PE(46:2)) were temporarily eliminated from the analysis, multiple regression analysis in men revealed a significant correlation of BAT-d with BMI. These results were consistent with those of many studies that used ^18^FDG-PET/CT, regardless of the presence of cold exposure [6, 14–17, 19, 20].

In vitro, androgens disrupted the thermogenic capacity of BAT. Furthermore, androgen deprivation caused the appearance of multilocular adipocytes within WAT depots, resulting in unique mRNA expression profiles in BAT [28]. However, in animal models, testosterone may induce weight loss through a combination of decreased energy intake and increased the resting metabolic rate with or without changes in BAT activity [29–32]. Furthermore, in in vivo androgen receptor-knockout models, BAT activity and energy expenditure were decreased [32–34]. Taken together, our findings suggested that androgens had positive effects on the observed increases in BAT-d in this study, consistent with previous animal studies. Moreover, if testosterone enhances irisin and promotes browning of irisin in WAT [35], we could hypothesize that testosterone may stimulate increased BAT formation or browning [32].

We did not find any effects of androgens on BAT-d in women, possibly because androgens are essential for normal adipogenesis in males and can impair essential adipocyte functions in nonhuman female primates [28]. Although we did not find a correlation between parameters of body adiposity and BAT-d in men, which has generally been observed in many studies [5, 8, 36], or in women in this study, the correlation of androgens with BAT-d remained in men. A previous study revealed a significant decrease in BAT-d with aging only in men, not in women, aged 20–85 years [8]; therefore, androgens, which usually show decreased concentrations with aging in men, may have confounded the correlations of body adiposity with BAT-d in previous studies [5, 8, 36].

17-β estradiol (E2) and progesterone enhance noradrenaline-induced lipolysis and therefore promote BAT function by modulating β1/β2-, α2A-, and β3-adrenergic receptor [37, 38]. However, we failed to find a relationship between BAT-d and these metabolites in women. PE is a key component, together with phosphocholine (PC), making up membrane lipids and determining the fate of adipocyte structure and function. Increases in the relative amounts of PE on the surfaces of lipid droplets can promote fusion of smaller droplets into larger ones, a characteristic of WAT [39], and the increases in plasma PE found in this study may indicate a reduction in PE concentration on the surface of lipid droplets, thereby conferring the adipocytes with BAT characteristics. BAT activity and/or volume are related to some lipid metabolites, such as LysoPC-acyl(C16:0), in humans [16]; however, this result was not confirmed in our current study. Instead, we found that although the relationship between BAT-d and PE disappeared after multiple regression analysis, individuals with greater adiposity had lower BAT-d values, which coincided with lower plasma PE concentrations, consistent with a previous study demonstrating a reduction in PE in the BAT after consumption of a high-fat diet in mice [12].

Recently, a study in mice reported that cold exposure for 1 month stimulated de novo synthesis of monomethyl branched-chain fatty acids (mmBCFAs) via mitochondrial branched-chain amino acid (BCAA) catabolism in BAT [40]. This result indicated that understanding the factors regulating mmBCFA physiology is essential for elucidating their functions and contributions to the biological importance of the BCAA catabolic pathway. In fact, on cold exposure, BAT actively utilizes BCAAs in the mitochondria for thermogenesis and promotes systemic BCAA clearance in mice and humans [41]. In turn, a BAT-specific defect in BCAA catabolism attenuates systemic BCAA clearance, contributing to the improvement of metabolic health. Thus, further research should be conducted to examine the associations of BCAA metabolism and seasonal changes in human BAT activity under normal living conditions in humans.

Lipidomic metabolites, which were correlated with BAT-d in this study, also show correlations with the amount of WAT. After we examined the relationships between lipidomic metabolites and indicators of WAT, such as %FAT and VFA, several new metabolites, including Cer(18:1/24:0), 3α,7α-dihydroxy-5β-cholestabonyl-CoA, and LysoPG(p-24:5), were found. Cer(18:1/16:0)/Cer(18:1/24:0) and Cer(18:1/22:0)/Cer(18:1/24:0) are related to increased risk of cardio-arterial disease-related death [42], indicating a reduction in risk by increasing Cer(18:1/24:0). Thus, our observation of the relationship between Cer(18:1/24:0) and %FAT, not VFA, was consistent with this previous study [42]. 3α,7α-dihydroxy-5β-cholestabonyl-CoA is involved in production of chenodeoxycholic acid, a bile acid, from 3α,7α-dihydroxy-5β-cholestanoic acid [43]. The negative correlations of this enzyme and LysoPC(p-24:5) with VFA found in men and women, respectively, indicated their effects on reducing the risk of metabolic syndrome.

Because it is difficult to conduct PET studies owing to ionizing radiation exposure and difficulties in monitoring the amount of cold exposure for each individual during daily life, we attempted to identify biomolecules in the blood associated with human BAT-d. We aimed to detect small amounts of biomolecules in the blood stream spilled over from the BAT. In fact, plasma LysoPC-acyl(C16:0) was reported to be associated with BAT activity in men [16]. However, plasma biomolecules may not modulate BAT activity, but could merely be a source of related metabolites released from the BAT. Although we carefully selected the locations of NIR_TRS_ measurements, [total-Hb]_sup_ may have been influenced by WAT, muscle tissue, and connective tissue in the area. However, our previous work revealed significant relationships between [total-Hb]_sup_ under thermoneutral conditions and cold-induced ^18^FDG-PET/CT parameters in the supraclavicular region (*r* = 0.74), but not in the deltoid muscle region. We also confirmed a significant correlation between CIT, an indicator for BAT activity [17], and [total-Hb]_sup_ in winter. Furthermore, a longitudinal study reported that [total-Hb]_sup_ increases with the ^18^FDG-PET/CT parameter during repeated thermogenic capsinoid intake, which is known to increase BAT activity and mass, and decreases after cessation of its intake [21]. Thus, we believe that NIR_TRS_ parameters, in particular [total-Hb]_sup_, can be used to evaluate BAT-d under thermoneutral conditions. In addition, we did not control the menstrual cycle for blood sampling. This may be why we failed to find a relationship between BAT-d and lipid metabolites in women. Further research on the effects of female hormones on BAT-d is needed in women.

In conclusion, certain lipids in plasma showed unique correlations with BAT-d depending on sex and season. Multivariate regression analysis in winter revealed a significant correlation between BAT-d and androgens only in men. Thus, androgens might be biomarkers which involved in activating BAT in men in the winter, but further research is needed to find some biomolecules in women.

## Supplementary information

Figure 1S. Relationships between metabolites and brown adipose tissue density (BAT-d) in summer

Figure 1S. Relationships between metabolites and brown adipose tissue density (BAT-d) in summer

Figure 2S. Relationships between phosphatidylethanolamine (PE(46:2)) and brown adipose tissue density (BAT-d)

Figure 3S. Relationships between diacylglycerol (DG(36:1)) and brown adipose tissue density (BAT-d)

Figure 4S. Interactions of brown adipose tissue density (BAT-d; high versus low), season, and sex

Table 1S. Liquid chromatography-time-of-flight-mass spectrometry conditions

Table 2S. Compounds that were significantly (*P* < 0.05) correlated with brown adipose tissue density

Table 3S. Correlations between body adiposity parameters and metabolites that remained significantly (*Q* < 0.05) correlated with brown adipose tissue density or body adiposity

Table 4S. Correlations between BAT density (BAT-d) in winter and season-dependent changes in metabolite levels (winter to summer)
