# Supplementary material for: Relationships between plasma lipidomic profiles and brown adipose tissue density in humans
Source: Int J Obes (Lond). 2020 Mar 3;44(6):1387–96. doi: 10.1038/s41366-020-0558-y (PMC7260127; doi:10.1038/s41366-020-0558-y)
Supplement: Supplementary file 1 — Figure 1S. Relationships between metabolites and brown adipose tissue density (BAT-d) in summer [file 41366_2020_558_MOESM1_ESM.docx]

**Supplemental information**

**Figure 1S.** Relationships between plasma metabolites and brown adipose tissue density (BAT-d) in summer. a) In summer, among metabolites showing correlations (*P* < 0.05) with BAT-d, phosphatidylethanolamine (46:2) (PE(46:2), labeled as 55 in Table 2S) and diacylglycerol (36:1) (DG(36:1), labeled as 93 in Table 2S) showed significant positive and negative correlations at Q < 0.05 in men, respectively. b) In women, no metabolites showed any significant correlations at Q < 0.05. *P* values were corrected by false discovery rates to yield Q-values, for considering independent multiple tests. The levels of BAT-d are shown in the right edge of the heat map. The units of the color scale were calculated dividing the value of each substance by the average of values of corresponding substances for men and women both in summer and winter. For example, yellow indicates the greater relative value of each metabolite, whereas blue indicates the lower relative value of each metabolite. The number of metabolites indicated below the heat map represents each substance, as listed in Table 2S. r, correlation coefficient between BAT-d and each metabolite.

**Figure 2S.** Relationships between phosphatidylethanolamine (PE(46:2)) and brown adipose tissue density (BAT-d). a) Relationships in men. b) Relationships in women. *P* values were corrected by false discovery rate to yield Q-values considering independent multiple tests.

**Figure 3S.** Relationships between diacylglycerol (DG(36:1)) and brown adipose tissue density (BAT-d). a) Relationships in men. b) Relationships in women. *P* values were corrected by false discovery rate to yield Q-values considering independent multiple tests.

**Figure 4S.** Interactions of brown adipose tissue density (BAT-d; high versus low), season, and sex. Three-way analysis of variance was used to test the interactions (BAT-d group × season × sex) and main effects (BAT-d group, season, and sex). To divide BAT-d into two groups (high and low), we used the cutoff value reported in a previous study in winter (17) (high, [total-Hb]_sup_ ≥ 74.0 μM; low < 74.0 μM). To conduct the analyses, categorical variables were set at “0” for the low group and “1” for the high group, “0” for summer and “1” for winter, and “0” for women and “1” for men. There was an interaction between BAT-d group (high versus low) and sex, but not an interaction between BAT-d group (high versus low) and season.

**Table 1S.** Liquid chromatography-time-of-flight-mass spectrometry conditions.

Notes: The mobile phase consisted of solvent A (0.5 mM ammonium formate in water, methanol, and acetonitrile at a volume ratio of 3:1:1) and solvent B (0.5 mM ammonium formate in 2-propanol).

**Table 2S.** Compounds that were significantly (*P* < 0.05) correlated with brown adipose tissue density.

**Table 3S.** Correlations between body adiposity parameters and metabolites that remained significantly (Q < 0.05) correlated with brown adipose tissue density or body adiposity in men or women or in winter or summer.

Notes: *P* values were corrected by false discovery rate to yield Q-values considering independent multiple tests. %BF: percentage of body fat; VFA: visceral fat area; androgens: testosterone, androstanedione, dehydroandrosterone, dehydroepiandrosterone, or epitestosterone; DG: diacylglycerol; PE: phosphatidylethanolamine; Cer: ceramide; DHCA-CoA: 3α, 7α-dihydroxy-5β-cholestanoyl-CoA; LysoPG: lysophosphatidylglycerol.

**Table 4S.** Correlations between BAT density (BAT-d) in winter and season-dependent changes in metabolite levels (winter to summer).

Notes: Spearman correlation analysis was used to evaluate the correlations among BAT-d in winter and metabolite levels (winter to summer) that remained significantly correlated with BAT-d or body adiposity in men or women or in winter or summer. Androgens: testosterone, androstanedione, dehydroandrosterone, dehydroepiandrosterone, or epitestosterone; DG: diacylglycerol; PE: phosphatidylethanolamine; Cer: ceramide; DHCA-CoA: 3α, 7α-dihydroxy-5β-cholestanoyl-CoA; LysoPG: lysophosphatidylglycerol.
