# Supplementary figures and images for: Relationships between plasma lipidomic profiles and brown adipose tissue density in humans
Source: Int J Obes (Lond). 2020 Mar 3;44(6):1387–96. doi: 10.1038/s41366-020-0558-y (PMC7260127; doi:10.1038/s41366-020-0558-y)

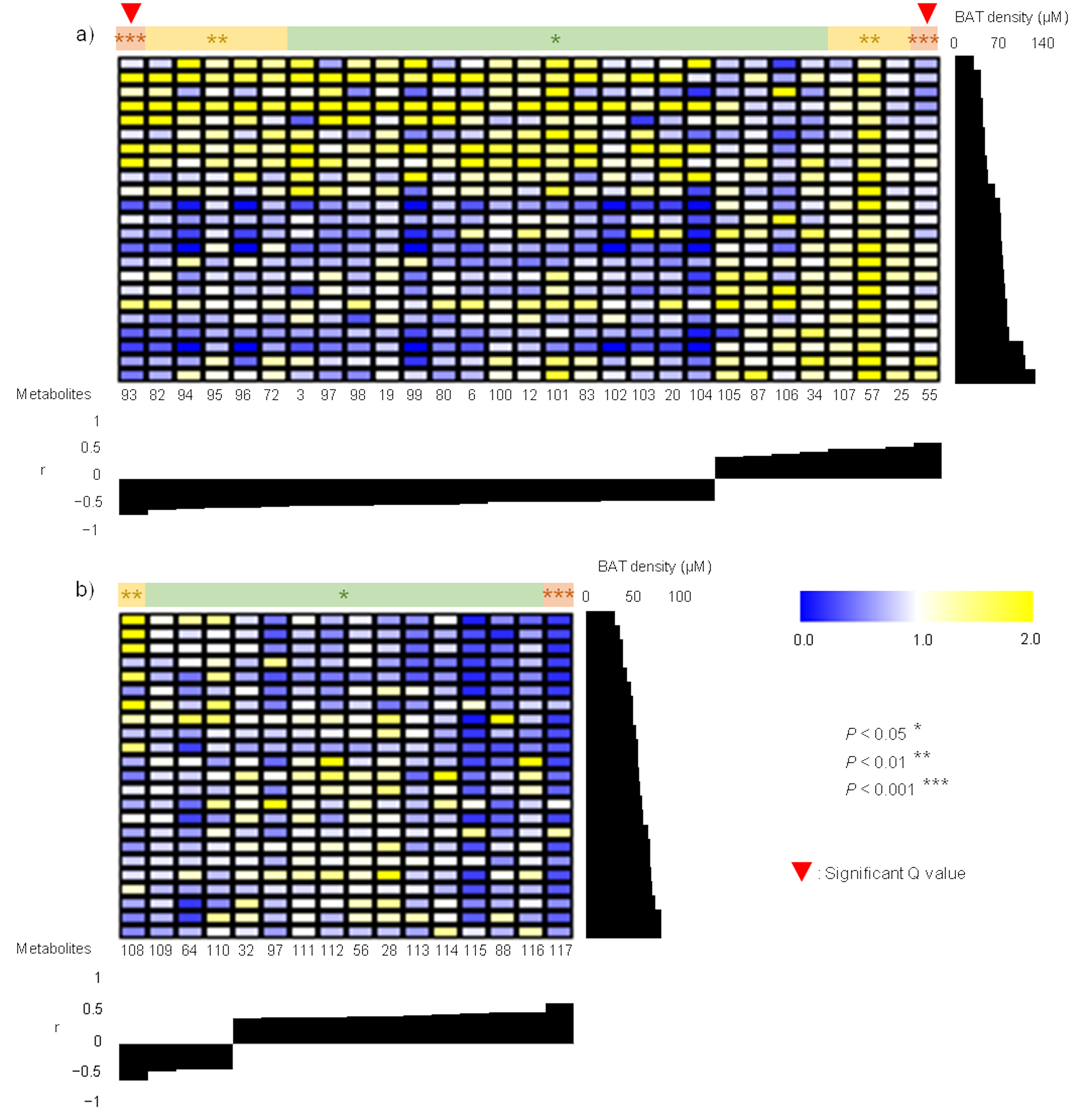

Supplement: Supplementary file 2 — Figure 1S. Relationships between metabolites and brown adipose tissue density (BAT-d) in summer [file 41366_2020_558_MOESM2_ESM.tif]

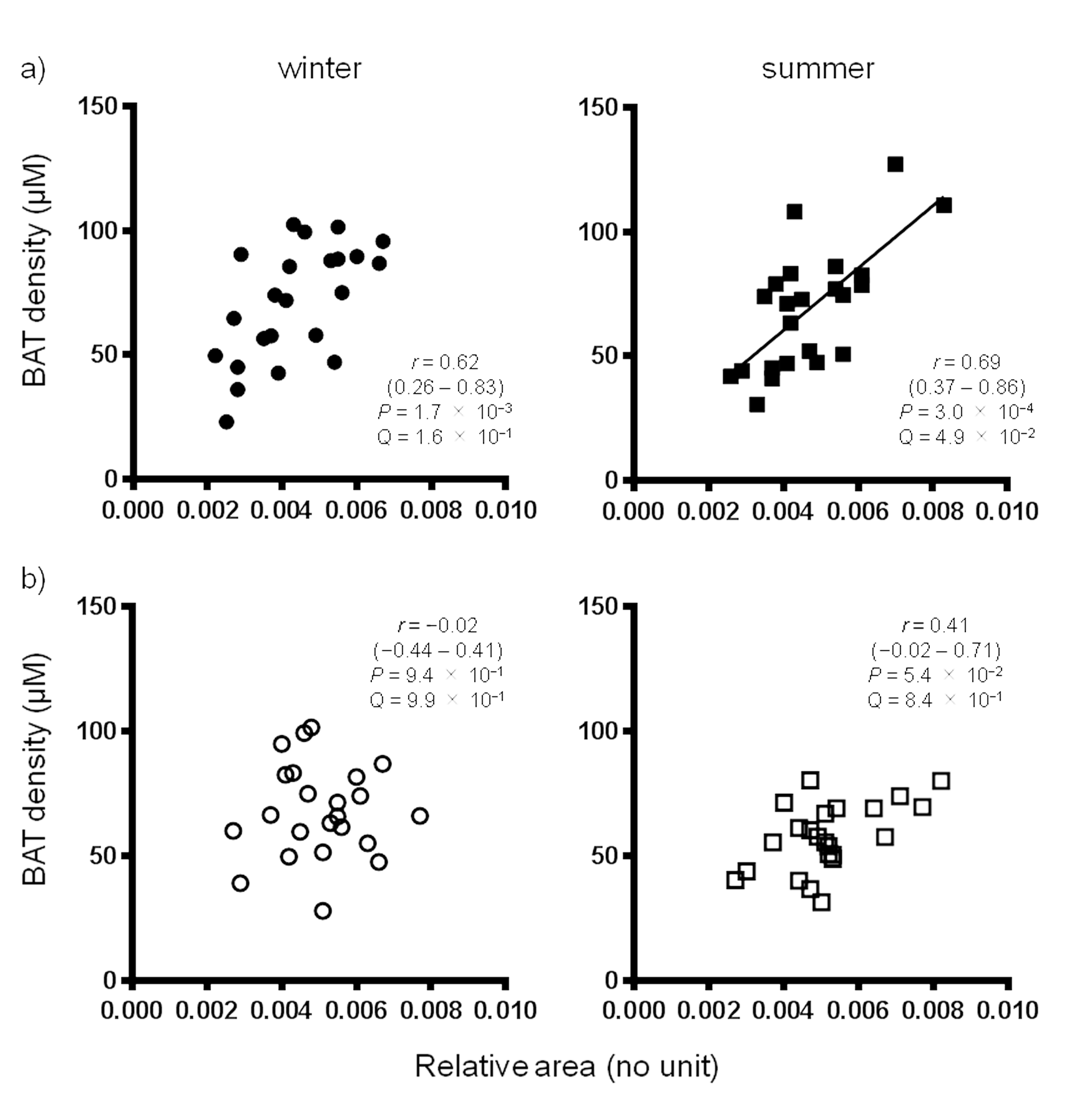

Supplement: Supplementary file 3 — Figure 2S. Relationships between phosphatidylethanolamine (PE(46:2)) and brown adipose tissue density (BAT-d) [file 41366_2020_558_MOESM3_ESM.tif]

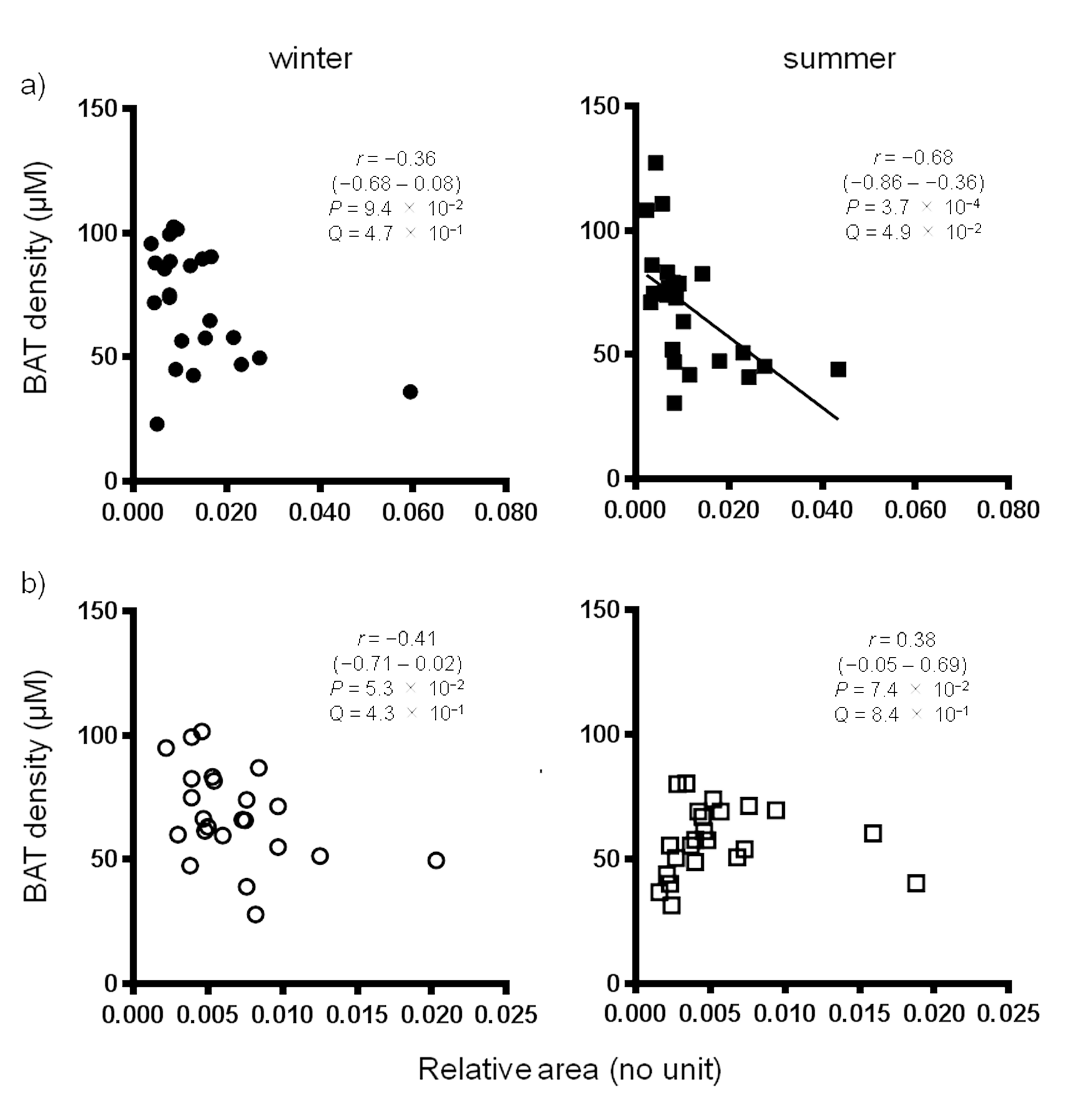

Supplement: Supplementary file 4 — Figure 3S. Relationships between diacylglycerol (DG(36:1)) and brown adipose tissue density (BAT-d) [file 41366_2020_558_MOESM4_ESM.tif]

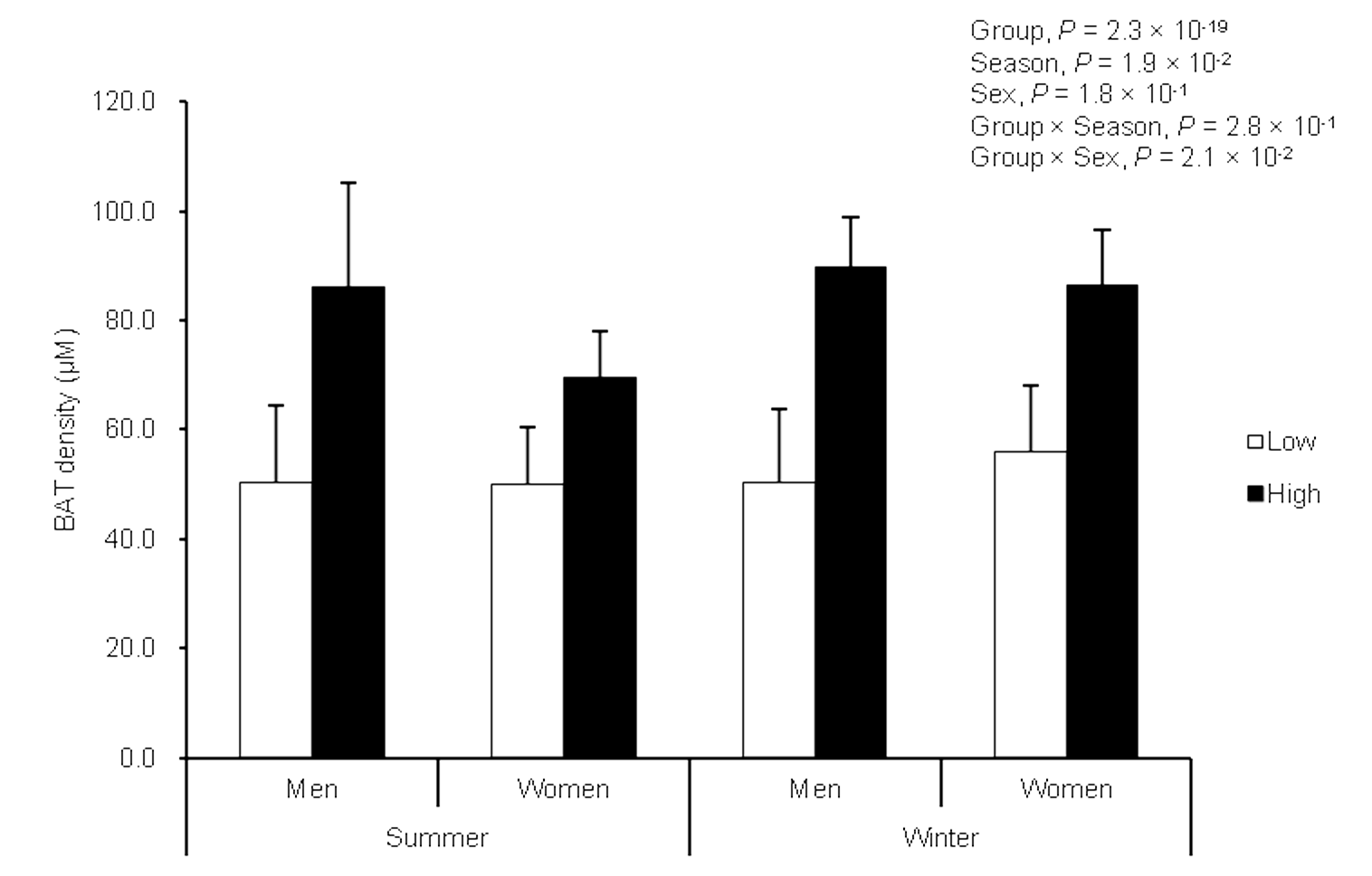

Supplement: Supplementary file 5 — Figure 4S. Interactions of brown adipose tissue density (BAT-d; high versus low), season, and sex [file 41366_2020_558_MOESM5_ESM.tif]
